# Carbapenemase-Producing Klebsiella pneumoniae Infections in Diabetic and Nondiabetic Hospitalized Patients

**DOI:** 10.7759/cureus.52468

**Published:** 2024-01-17

**Authors:** Athira Pattolath, Prabha Adhikari, Vidya Pai

**Affiliations:** 1 Department of Geriatric Medicine, Yenepoya University Medical College Hospital, Mangalore, IND; 2 Department of Microbiology, Yenepoya University Medical College Hospital, Mangalore, IND

**Keywords:** carbapenemase, diabetes, carbapenem resistance, antimicrobial resistance, klebsiella pneumoniae

## Abstract

Background: Carbapenem-resistant Klebsiella pneumoniae (CRKP) infection has recently attracted widespread attention due to its limited treatment options and significant morbidity and mortality rates. This study aimed to examine the relationship between risk factors and antimicrobial resistance in individuals with and without diabetes for the development of carbapenemase-producing K. pneumoniae infections.

Methods: Between May 2019 and January 2021, a prospective study involving patients with and without diabetes who were infected with K. pneumonia, was carried out in a tertiary care hospital. Six hundred K. pneumoniae isolates were collected from various clinical samples, such as pus/wound samples, urine, respiratory samples, blood, and body fluids. An antimicrobial susceptibility test in K. pneumoniae was performed and compared between diabetics and nondiabetics. Univariate and multivariate logistic regression were used to identify independent risk factors for K. pneumoniae infections in the diabetic group and nondiabetic group separately. Multiplex PCR was used to detect genes that produce carbapenemase.

Results: A total of 600 patients were infected with K. pneumoniae, with 300 (50%) being diabetic and 300 (50%) being nondiabetic. We found that diabetics had higher antimicrobial resistance to numerous routinely used drugs for infection than the nondiabetic group. In the multivariate analysis of the variables, it was found that immunosuppressive therapy, prior antibiotic use, mechanical ventilation, and urinary catheter use were all significant risk factors influencing the development of K. pneumoniae infections in diabetic patients. Diabetics had a higher prevalence of carbapenemase-producing K. pneumoniae than nondiabetics. Outcome measures in K. pneumoniae patients revealed that the diabetic group had considerably higher infection-related mortality.

Conclusion: We found that CRKP infection was associated with higher resistance to antibiotics in the diabetic group. Furthermore, the diabetic group had a higher prevalence of carbapenemase-producing K. pneumoniae than the nondiabetic group. Crucially, in order to lower mortality without worsening antibiotic resistance and metabolic damage, more focus has to be placed on sensible and efficient antibiotic and supportive care therapies.

## Introduction

Type 2 diabetes mellitus (T2DM), which has an enormous impact on morbidity, mortality, disability, and economic expenditures, is a global health issue that disproportionately affects low and middle-income countries (LMICs) [1-3]. While most T2DM research has concentrated on its causes, effects, and complications concerning noncommunicable diseases, T2DM has recently received more attention as a risk and prognostic factor for infectious communicable diseases [4-6]. Even though antibiotic resistance causes a significant illness burden globally and in LMICs, research on it is still lacking in this sector, which combines T2DM and infectious diseases [7-9].

A serious public health issue associated with a number of infections is antimicrobial resistance to a broad spectrum of bacterial pathogens. The resistance pattern of microorganisms is always changing, and unregulated antibiotic usage, along with careless attitudes in society towards medication, poses an immense risk to global health. Among people with diabetes, infections are a rather common reason for hospital stays or doctor visits. Compared to people without diabetes, people with diabetes may be more susceptible to infections. Because of their higher blood glucose levels, weakened immune systems, neuropathy, and reduced blood supply to their extremities, diabetics are more susceptible to infections and wounds that heal slowly. Numerous bacteria can lead to infections in diabetes people [10,11].

Carbapenem, a class of antibiotic medicines that includes imipenem, meropenem, and ertapenem, is the standard treatment for severe infections caused by K. pneumoniae, a well-known opportunistic pathogen. However, carbapenem-resistant K. pneumoniae (CRKP) has already become a serious concern to human health. The World Health Organisation (WHO) designated CRKP as Priority 1 (critical priority) in the worldwide priority list of antimicrobial-resistant bacteria in 2018 [12]. The proportion of K. pneumoniae infections that are resistant to carbapenems is on the rise and has reached 50% in some regions of Europe and the Eastern Mediterranean. Since carbapenems are typically the last line of defense against significant K. pneumoniae infections, CRKP infection is challenging to treat. Nearly all existing treatments are ineffective against K. pneumoniae because of the presence of carbapenemase-producing genes that lead to carbapenem resistance. Mortality rates in people infected with CRKP in various parts of the world range from 33% to 50%, which is much greater than the death rate caused by CSKP infection [13,14]. Considering the substantial burden of type 2 diabetes, its possible role as a risk and predictive factor for infectious diseases, and the global issue of antibiotic resistance, we need to carefully examine whether people with T2DM are more likely to get infections caused by resistant bacteria.

This information may help in directing empirical treatment, which would benefit T2DM patients by allowing them to recover more quickly from infections and by minimizing the prevalence of antibiotic-resistant bacteria by recommending more precise treatments [11].

Nevertheless, there is a dearth of conflicting evidence regarding K. pneumoniae antibiotic resistance in pneumonia or other infections. As far as we know, there has only been one study that looked at the differences between those with diabetes and those without diabetes in terms of the risk factors for pneumonia mortality [15]. Despite the adverse impacts of antibiotics on metabolism and the significant risk of developing K. pneumoniae in diabetics, it is critical to comprehend antimicrobial resistance and the elements that contribute to death in this risk group. Our study aimed to conduct a comparatively thorough investigation into antibiotic resistance and the existence of carbapenenemase-generating genes in K. pneumoniae patients with and without diabetes.

## Materials and methods

Between May 2019 and January 2021, a prospective study involving patients with and without diabetes who were infected with K. pneumoniae was carried out in a tertiary care hospital. The study was conducted after receiving approval from the Institutional Ethics Committee Board (YEC-1 2019/038, dated 11-04-2019). Our analysis only included the first positive K. pneumoniae culture from each patient's clinical sample.

Clinical data collection and risk factor analysis

Patients with and without diabetes were separated into two groups. The study included 300 diabetics and 300 nondiabetic adult hospitalized patients with K. pneumoniae. According to the American Diabetes Association (ADA) 2020 standards, a diagnosis of diabetes was determined when the patient's fasting blood glucose level was 126 mg/dL or their glycosylated hemoglobin (HbA1c) level was 6.5%. Diabetes mellitus duration and type are documented. If K. pneumoniae was detected 48 hours after admission, medical records were reviewed and gathered. The following information was used to support the analysis: demographics; a history of hospitalization within the previous six months; antibiotic therapy administered in the 30 days prior to the positive culture; a recent surgical procedure; and recent invasive procedures as well as co-morbidities. Mortality, discharged against medical advice (DAMA), sepsis, length of stay in the ICU, evaluation of SOFA score in ICU patients, and length of mechanical ventilation were all monitored for each patient.

Antibiotic susceptibility testing

Bacterial susceptibility to antimicrobial drugs was assessed in vitro using the standard Kirby-Bauer's disc diffusion method (MHA) plates, as suggested by the Clinical Laboratory Institute (CLSI) [16]. Ampicillin, amoxicillin-clavulanic acid, amikacin, ciprofloxacin, ceftazidime, cefotaxime, chloramphenicol, tetracycline, colistine, imipenem, meropenem, piperacillin, piperacillin-tazobactam, aztreonam, chloramphenicol, gentamycin, and levofloxacin were all tested. The Escherichia coli strain ATCC25922 was chosen as a quality control strain.

MIC determination using the broth microdilution method

The broth microdilution method was used in accordance with CLSI guidelines. A 0.5 McFarland suspension of the isolates was prepared and diluted 100-fold with cation-adjusted Mueller-Hinton broth (CAMHB). Then, 50 µL of the bacterial solution was seeded into a 96-well plate, along with 50 µL of CAMHB and serial antibiotic concentrations. In the final inoculum, there were approximately 5x10^5^ CFU/mL of bacteria. The suspension was cultured at 35°C for 18-20 hours [16].

Carba NP test for phenotypical detection of carbapenemase production

After adding API suspension media (25 µL; bioMérieux, New Delhi, India) to the wells, five to six colonies from the newly cultured culture plate were placed in the allocated well. The turbidity of the inoculum was contrasted with that of the supplied strip. Next, two wells were filled with 10 µL of inoculum, one of which contained imipenem. Gram-negative bacteria that produced metallo β lactamases (MBL) were treated with imipenem as the carbapenemase zinc substrate; positive results were defined as those in which the color of the bacteria changed from red to yellow, orange, or thick orange in comparison to the control well [17-19].

Detection of carbapenemase genes

HiPurA® Genomic DNA Purification kits (Hi-Media®, India) were used to extract DNA. These kits utilize Miniprep spin columns' speed and adaptability, as well as the enhanced silica gel membrane's reversible nucleic acid binding characteristics to produce high-quality DNA. The carbapenemase gene (multiplex) probe-based Hi-Media Hi-PCR kit was used to locate particular regions of the gene that encode the carbapenemase enzyme, such as blaIMP, blaOXA-48, blaVIM, and bla NDM-1. Positive, internal, and negative controls were used in accordance with the company's guidelines. The primers and probes for blaKPC, blaOXA-48, blaIMP, and blaNDM are displayed in Table 1.

**Table 1:** Primers and probes used in the multiplex, real-time PCR test

Gene	Primer Names	Probe	Sequences (5'-3')	Wavelength (nm)
blaNDM	NDM Carbapenemase	FAM labeled hydrolysis probe	F:GGTTTGGCGATCTGGTTTTC R:CGGAATGGCTCATCACGATC	FAM (465–510)
blaKPC	KPC Carbapenemase	HEX labeled hydrolysis probe	F:ATGTCACTGTATCGCCGTCT R:TTTTCAGAGCCTTACTGCCC	610 (540–610)
blaIMP	IMP Carbapenemase	Texas Red labeled hydrolysis probe	F:GAATAGRRTGGCTTAAYTCTC R:CCAAACYACTASGTTATC	640 (610–645)
blaOXA	OXA-48 Carbapenemase	Texas Red labeled hydrolysis probe	F:TTGGTGGCATCGATTATCGG R:GAGCACTTCTTTTGTGATGGC	580 (540–580)
blaVIM	VIM Carbapenemase	Cyan500 labeled hydrolysis probe	F:GTTTGGTCGCATATCGCAAC R:AATGCGCAGCACCAGGATAG	FAM (465–510)

A 25 µL reaction mixture, including 5 µL of sample DNA and 20 µL of master mix, was used for this experiment. The master mixes were made in accordance with Table 2's instructions.

**Table 2:** Composition of the master mix for detecting carbapenemase gene

Components	Product Code	The quantity that needs to be included for a 25 µL reaction (1R)	
		CRG1	CRG2
Hi-Quanti 2X Realtime PCR Master Mix	MBT180	12.5 µL	12.5 µL
CRG1 Primer-Probe Mix	DS0949	4 µL	-
CRG2 Primer-Probe Mix	DS0950	-	4 µL
Internal Control Primer-Probe Mix	DS0498	1 µL	1 µL
Internal Control B DNA	DS0385A	1 µL	1 µL
Molecular Biology Grade Water for PCR	ML065	1.5 µL	1.5 µL
Positive Control/Negative Control/Template DNA	-	5 µL	5 µL
Total Volume	-	25 µL	25 µL

The SLAN-96P real-time PCR system was used for the cycling method, which included a preliminary denaturation step of 10 minutes at 95°C, a further denaturation step of 5 seconds at 95°C, 45 cycles of annealing and extension lasting one minute each, and a final holding stage. Cycle threshold values greater than ≤40 were regarded as positive. The kit enables the accurate and precise detection of carbapenemase encoding genes that are present singly or in combination in single tube reactions [19,20].

Statistical evaluation

Frequencies and percentages were provided for categorical variables. Using univariate analysis, the major risk factors for developing CRKP among diabetic and nondiabetics were identified. A P-value of 0.5 was chosen as the level of significance to test the null hypothesis. In order to identify independent risk factors for CRKP infection among diabetics, stepwise multiple logistic regression analyses of the risk factors were also calculated for variables found significant by conventional statistical analysis. All of the data were analyzed with Statistical Product and Service Solutions (SPSS) (version 23.0; IBM SPSS Statistics for Windows, Armonk, NY).

## Results

A total of 600 patients were infected with K. pneumonia, among the sample size 300 (50%) isolates being diabetic and 300 (50%) being nondiabetic. The isolates were collected from various clinical samples, such as pus/wound samples, urine, respiratory samples, blood, and body fluids. Table 3 displays the sample-wise distribution of K. pneumoniae isolates from the diabetes and nondiabetic groups.

**Table 3:** Source-wise distribution of K. pneumoniae isolates among diabetics and nondiabetics patients Data presented as n (%)

Clinical samples (N)	Diabetic group (N=300)	Nondiabetic group (N=300)
Pus/Wound swab	100 (33.3%)	140 (46.6%)
Sputum	75 (25%)	109 (36.3%)
Urine	96 (32%)	48 (16%)
Blood	23 (7.7%)	3 (1%)
Body fluids	6 (2%)	0

In the diabetic group, the male gender predominated (68%), and the average age was 64.23±6.7 years. The mean HbA1c level was 9.3±2.7; the diabetic duration was 7.0±7.4 years. When compared to the nodiabetic group, the diabetic group had an elevated mass index (BMI) (26.4 vs. 21.8), were older in age (68.5 vs. 62.7 years), and had more comorbid disorders, such as hypertension (47% vs. 14%) and cardiovascular diseases (52% vs. 10%). Furthermore, diabetic patients had a greater rate of past hospitalization, prior surgery, and usage of immunosuppressive medicines.

Key risk factors identified by univariate analysis included hypertension (P=0.000), cardiovascular disease (P=0.000), chronic kidney disease (P=0.000), use of immunosuppressive therapy (P=0.000), respiratory illness (P=0.033), prior antibiotic exposure (P=0.000), and prior surgery (P=0.001). The use of indwelling devices, such as urinary catheters, was also found to be significantly (P=0.000) linked with K. pneumoniae infection in diabetics by the univariate analysis. Table 4 shows the results of a univariate examination of clinical features in K. pneumoniae-infected the diabetic group and nondiabetic group.

**Table 4:** Clinical features and univariate analysis of K. pneumoniae patients with and without diabetes

Variables	Diabetics (300)	Nondiabetics (300)	OR	P-value
Smoking	15 (5%)	16 (5%)	0.934 (0.453-1.926)	0.854
Alcohol	17 (6%)	13 (4%)	1.326 (0.632-2.781)	0.454
Hypertension	140 (47%)	43 (14%)	5.230 (3.524-7.760)	0.000
Chronic obstructive pulmonary disease	52 (17%)	44 (15%)	1.220 (0.787-1.890)	0.373
Asthma	26 (9%)	33 (11%)	0.768 (0.447-1.319)	0.337
Tb	10 (3%)	12 (4%)	0.828 (0.352-1.946)	0.664
Immunosuppression	136 (45%)	20 (7%)	11.610 (6.990-19.284)	0.000
Chronic kidney diseases	209 (70%)	13 (4%)	50.704 (27.610-93.117)	0.000
Chronic liver disease	28 (9%)	12 (4%)	2.471 (1.231-4.957)	0.009
Cardiovascular disese	155 (52%)	29 (10%)	9.989 (6.402-15.586)	0.000
Pulmonary disease	146 (49%)	172 (57%)	0.706 (0.511-0.973)	0.033
Neurological disease	33 (11%)	44 (15%)	0.719 (0.444-1.165)	0.179
Autoimmune disease	11 (4%)	14 (5%)	0.778 (0.347-1.742)	0.540
Prior history of surgery details	291 (97%)	72 (24%)	102.389 (50.123-209.157)	0.000
Tumour	19 (6%)	22 (7%)	0.854 (0.452-1.614)	0.627
Use of broad-spectrum antibiotics	152 (51%)	65 (22%)	3.713 (2.601-5.300)	0.000
Previous hospitalisations	220 (73%)	80 (27%)	7.563 (5.266-10.860)	0.000
Central venous catheter	59 (20%)	53 (18%)	1.141 (0.756-1.721)	0.530
Urinary catheter	241 (80%)	94 (31%)	8.952 (6.153-13.024)	0.000
Nasogastric	178 (59%)	185 (62%)	0.907 (0.654-1.258)	0.559
Parentral nutrition	138 (46%)	142 (47%)	0.948 (0.688-1.306)	0.743
ICU admission	60 (20%)	40 (13%)	1.625 (1.050-2.515)	0.028
ICU stays more than one week	53 (18%)	29 (10%)	2.005 (1.235-3.255)	0.004
Sofascore >12	42 (14%)	30 (10%)	1.465 (0.890-2.412)	0.132
Septic shock	42 (14%)	20 (7%)	2.279 (1.3.4-3.985)	0.003
Mechanical ventilation	53 (18%)	29 (10%)	2.005 (1.235-3.255)	0.004
Recovered	122 (41%)	271 (90%)	0.073 (0.047-0.115)	0.000
DAMA (discharged against medical advice)	98 (33%)	22 (7%)	6.131 (3.731-10.074)	0.000
Death	80 (27%)	7 (2%)	15.221 (6.894-33.607)	0.000

When multivariate analysis was applied to the factors, immunosuppressive therapy (P=0.000), mechanical ventilation (P=0.013), prior exposure to antibiotics (P=0.000), and use of a urinary catheter (P=0.000) were all found to be significant risk factors affecting the development of K. pneumoniae infections among diabetic patients. The multivariate logistic regression analysis's findings regarding the influence of risk factors on people with K. pneumoniae infection among the diabetic group and nondiabetic group are shown in Table 5.

**Table 5:** Multivariate analysis of K. pneumoniae patients with and without diabetes

Variables	Odds ratio	95% Confidence Interval	P-value
Immunosuppression	24.769	7.303-84.004	0.000
Mechanical ventilation	3.022	1.257-7.268	0.013
Use of broad-spectrum antibiotics	0.120	0.049-0.296	0.000
Hypertension	0.319	0.089-1.139	0.079
Urinary catheter	5.237	2.510-10.929	0.000
Death	2.653	0.005-7.071	0.051

According to outcome measures of the study in K. pneumoniae patients, the infection-related mortality was considerably greater in diabetic patients (27%) as opposed to nondiabetic patients (2%), and the diabetic group had a greater rate of mechanical breathing and longer ICU stays.

Based on antimicrobial susceptibility, in the diabetic group of 300 samples, 234 (78%) were infected with CRKP isolates, and 66 (22%) were infected with CRKP isolates. In the nondiabetic group of 300 samples, 116 (39%) were infected with CRKP isolates, while 184 (61%) were infected with CRKP isolates. Table 6 lists the distribution of K. pneumoniae isolates among the diabetic group and nondiabetic group.

**Table 6:** Distribution of K. pneumoniae isolates among the diabetic and nondiabetic groups Abbreviations: CRKP, Carbapenem-resistant K. pneumoniae isolates; CSKP, Carbapenem-susceptible K. pneumoniae isolates; ESBL, Extended-spectrum β-lactamase; MDR, Multi-drug resistant

Variables	Diabetics (N=300)	Nondiabetics (N=300)	P-value
CRKP	234 (78%)	116 (39%)	0.000
CSKP	66 (22%)	184 (61%)	0.000
ESBL	241 (80.3%)	52 (17.3%)	0.000
MDR	215 (71.6%)	33 (11%)	0.000
Carbapenemase producers	229 (76.3%)	55 (18.3%)	0.000

**Figure 1:**
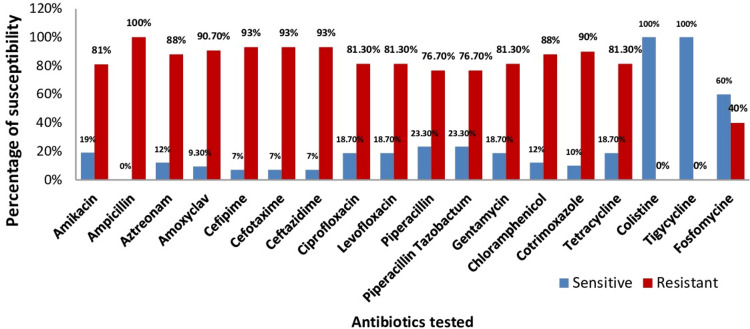
Antibiotic susceptibility pattern of K. pneumoniae isolates in diabetic patients The interpretation of the results was according to the Clinical and Laboratory Standards Institute (CLSI) 2020 guidelines.

The majority of the isolates of K. pneumoniae from diabetic patients exhibited high resistance to cefepime (93%), cefotaxime (93%), ceftazidime (93%), cotrimoxazole (90%), amoxyclav (90%), and chloramphenicol (88%). Ampicillin resistance was extremely high in all K. pneumoniae isolates. The isolated K. pneumoniae from nondiabetic patients was extremely sensitive to various antibiotics, in addition to its intrinsic ampicillin resistance. In the diabetes group, 71.6% of the isolates were MDRs, compared to 11% of the isolates in the nondiabetic group. Among the 18 drugs examined, K. pneumoniae demonstrated 40% resistance to fosfomycin and 100% sensitivity to tigecycline and colistine among the diabetic group. The antimicrobial resistance pattern of K. pneumoniae isolates in the diabetic group is shown in Figure 1.

There was no statistically significant difference in MIC for meropenem between the diabetic group and nodiabetic group. The majority of the CRKP isolates displayed MIC ranges of 4-8 µg/mL.

In the total 600 isolates of K. pneumoniae, 284 of them were carbapenemase-positive isolates. Among those carbapenemase producers, 229 (81%) isolates were from the diabetic group, while 55 (19%) were from the nondiabetic group. Among the 229 isolates of the diabetic group, 80 (35%) of the isolates tested positive for the blaNDM-1 gene, 65 (28.3%) for the blaOXA-48 gene, and 84 (36.6%) for both the blaOXA-48 gene and the blaNDM-1 gene. Among the 55 nondiabetic individuals, 25 (21.5%) isolates were positive for the blaNDM-1 gene, 19 (16.3%) isolates were positive for the blaOXA-48 gene, and 11 (9.4%) isolates were positive for both the blaNDM-1 and the blaOXA-48 genes. In the study, the whole sample data revealed the following: the mean Ct value of blaNDM-1 was 16, with a range of 12-24. The mean Ct value of the blaOXA-48 results ranged from 10 to 20. Table 7 displays the findings of the Ct values.

**Table 7:** Multiplex quantitative real-time PCR test for carbapenemase genes in CRKP isolates Shown are cycle threshold values (mean). Target genes for the PCR set are blaNDM, blaKPC, blaIMP, blaOXA, and blaVIM. Both blaNDM-1 and blaOXA-48-like are regarded as positive when the Ct value is less than 40.

Carbapenemase genes	No. of carbapenemase genes	C_t _value (mean)
blaNDM-1	105	16
blaOXA-48	84	15
blaIMP	0	Not detected
blaKPC	0	Not detected
blaVIM	0	Not detected

## Discussion

In this investigation, we sought to evaluate whether there are variations in antimicrobial resistance, risk factors for contracting K. pneumoniae infections, and carbapenemase production in diabetes and nondiabetic patients.

We found the following: (1) K. pneumoniae showed higher antimicrobial resistance to several commonly administered drugs in diabetics compared to nondiabetics. (2) Different risk factors for developing K. pneumoniae infections were found in diabetic patients compared to nondiabetics: immunosuppressive therapy, mechanical ventilation, prior antibiotic exposure, and use of a urinary catheter were all found to be significant risk factors. (3) Compared to nondiabetics, diabetics had a greater prevalence of carbapenemase-producing K. pneumoniae isolates.

According to the study, K. pneumoniae demonstrated significant antibiotic resistance among diabetes patients in our study. The majority of antibiotics in this diabetic group were resistant to the CRKP strains, which is probably related to the type and extent of antibiotics exposure and the presence of antibiotic-resistance genes [21]. Remarkably, our study found that K. pneumoniae had a higher rate of antibiotic resistance to the tested antibiotics than the Indian surveillance study reported [22]. Moreover, both diabetic and nondiabetic individuals show positive in vitro activity for tigecycline and colistin towards CRKP isolates; however, colistin resistance is a major public health concern that develops during therapy. It is mainly associated with genetic alterations in lipid metabolism [23,24].

A comprehensive study of antibiotic resistance and hospital mortality risk factors in K. pneumoniae patients with and without diabetes was carried out by Liu et al. [15]. According to their research, compared to nondiabetics, diabetics have reduced antimicrobial resistance of K. pneumoniae to many frequently prescribed drugs. Additionally, among hospitalized individuals with diabetes, there was a decreased incidence of CRKP in K. pneumoniae [15]. These variances in the susceptibility of bacteria to antibiotics, which fluctuate significantly between studies, may be explained by variations in bacterial ecologies and the circumstances under which antibiotics are used, both of which are still highly distinct.

The well-known practice of self-medication, the illegal sale and use of drugs, and the growth of covert healthcare facilities run by unqualified medical staff who frequently prescribe antibiotics in noncompliant doses and durations may all contribute to the extremely high resistance rates against beta-lactamines and quinolones found in our study. Additionally, our study found a link between antibiotic resistance and diabetes status. To combat the emergence of drug-resistant infections, research and interventions must focus on the senior diabetes population [25]. Our research confirmed findings from earlier studies that diabetics had higher BMIs, more co-morbidities, and older ages than nondiabetics [15,26]. Diabetes was found to be an independent risk factor for hypervirulent K. pneumoniae infection in a prior investigation [27]. According to Liu et al., CRKP was independently linked to higher mortality in BSI [15]. In the current investigation, it was discovered that CRKP was independently linked to higher mortality in diabetics.

Our investigation revealed a comparatively high hospital mortality rate for K. pneumonia. Furthermore, auto-discharge patients had a higher likelihood of unfavorable outcomes. Tian et al. discovered a 33.3% 28-day death rate in CRKP-BSI [28]. Our analysis of hospital mortality in CRKP pneumonia was comparable to their study, but mortality in CRKP infection was significantly higher. This difference could be explained by the fact that, in our study, CRKP primarily arose from lung infections, which were associated with a higher mortality rate.

Invasive mechanical ventilation was found to be an independent risk factor for diabetes in our study. This is presumably because diabetics experienced more pulmonary function impairment due to diffusion, as well as challenges with wound healing brought on by invasive manipulation. People with diabetes are typically older and have more renal and cardiovascular impairment. Therefore, compared to other risk factors or nondiabetics, diabetics may experience K. pneumoniae with a similar or even greater degree of severity. The carbapenemases genotypic detection revealed that 24% of the isolates had the blaOXA-48 gene and 30% of the isolates carried the blaNDM-1 gene.

The carbapenemases genotypic detection revealed that 29% of the isolates had the blaOXA-48 gene and 37% of the isolates carried the blaNDM-1 gene. These results align with those of Jaggi et al., who found that, while VIM and KPC were absent from all of their isolates, the NDM gene was found in 35.9% of CRKP isolates, either by itself or in conjunction with OXA-48 [29].

Limitations

Our discoveries in antimicrobial resistance and its mechanism require further validation in metabolomics, genomes, and a wider population, which is also the focus of our future research. At the same time, we compare the antimicrobial resistance of CRKP, which has lately become a hot topic, in different subgroups to make the results more trustworthy. Additionally, we evaluated the differences in K. pneumoniae infection risk variables between diabetics and nondiabetics, which was a poorly studied approach.

## Conclusions

We found that diabetics had higher antimicrobial resistance to numerous routinely used drugs for infection than nondiabetics. In the multivariate analysis of the variables, it was found that immunosuppressive therapy, prior antibiotic use, mechanical ventilation, and urinary catheter use were all significant risk factors influencing the development of K. pneumoniae infections in diabetic patients. Diabetics had a higher prevalence of carbapenemase-producing K.pneumoniae than nondiabetics. Outcome measures in K. pneumoniae-infected patients revealed that diabetes patients had considerably higher infection-related mortality. Most importantly, in order to lower mortality rates without worsening antibiotic resistance and metabolic damage, more focus has to be placed on sensible and efficient antibiotic and supportive care strategies.
